# The Potential Health Risk Associated with Edible Vegetables Grown on Cr(VI) Polluted Soils

**DOI:** 10.3390/ijerph17020470

**Published:** 2020-01-10

**Authors:** Richard Oruko Ongon’g, Joshua N. Edokpayi, Titus A. M. Msagati, Nikita T. Tavengwa, Grace N. Ijoma, John O. Odiyo

**Affiliations:** 1 Department of Ecology and Resource Management, University of Venda, Private Bag X5050, Thohoyandou 0950, South Africa; 2 Department of Hydrology and Water Resources, University of Venda, Private Bag X5050, Thohoyandou 0950, South Africa; Joshua.Edokpayi@univen.ac.za (J.N.E.); John.odiyo@univen.ac.za (J.O.O.); 3 Nanotechnology and Water Sustainability Research Unit, College of Science Engineering and Technology, University of South Africa, P.O. Box 392, UNISA 003, The Science Campus, Roodepoort 1709, Johannesburg, South Africa; msagatam@unisa.ac.za; 4 Department of Chemistry, University of Venda, Private Bag X5050, Thohoyandou 0950, South Africa; nikita.tavengwa@univen.ac.za; 5 Institute for Development of Energy for African Sustainability, College of Science, Engineering and Technology University of South Africa, 28 Pioneer Ave, Florida Park, Roodepoort 1709, Johannesburg, South Africa; nkechiijoma@gmail.com

**Keywords:** bioaccumulation, edible vegetables, hazard quotient, health index, speciation

## Abstract

This study reports on the assessment of the growth potential of five edible vegetables, which were grown in Cr(VI) spiked soils. The vegetable plants that were used in this study were *Vigna angularis*, *Cicer arietinum*, *Spinacia oleracea*, *Amaranthus dubius Thell* and *Phaseolus vulgaris*. Dried ground samples from roots, stems and leaves were analysed for various oxidation states of Cr. The daily intake of chromium, hazard quotient (HQ) and hazard index (HI) methods were employed to assess the potential human health risks posed by these Cr oxidation states through vegetable consumption. The results showed that *Vigna angularis* was the only vegetable that germinated in highly concentrated Cr(VI) in the simulated soil (456 mg/kg). The highest total chromium (Ch_T_) bioaccumulated in the roots was found in *Phaseolus vulgaris* at 0.8. The highest Ch_T_ translocation factor in the stem was that of *Cicer arietinum* and *Vigna angularis* at 0.30. The same plants translocated the highest Ch_T_ to the leaf at 0.7. A child or an adult consuming such contaminated *Cicer arietinum* vegetables were likely to take in between 508 and 785 mg/day of Ch_T,_ which are above the World Health Organisation guidelines of 220 and 340 mg/day, respectively. The highest HQ was found in *Cicer arietinum* at 8.7 and 13.4 for adults and children, respectively. The same species of plants also had high HI at 17.4 and 27.2 for adults and children, respectively. This indicated that consumers of the edible vegetables grown in Cr(VI) rich soils may be exposed to health risks, and the children were more likely to be vulnerable to these adverse effects than the adults.

## 1. Introduction

Generally, industrial activities tend to generate a wide range of wastes, which have the potential to impact the ecosystems negatively and also lead to high-costs of treatment when such wastes are discharged to unprotected environments [1]. In developing countries, environmental laws on waste management and waste disposal are either non-existent or ineffective where they do exist [2]. The use of soils from highly contaminated dumpsites as a soil improver to grow edible crops is a common practice in many developing countries. However, such a practice tends to cause bioaccumulation of toxic heavy metals in them. High concentrations of these metal pollutants in edible crops are known to be associated with potential health risks to consumers [3,4]. The contamination of foods is a problem that has the potential to affect populations far away from the place where such crops are grown through trade pacts or food aid [5].

Tannery effluents and solid wastes generated from the tanning process are known to pollute the environment with heavy metals and acids [5]. In developing countries, most of these wastes find their way into the environment through poor open dumping. Open dumping of solid tannery wastes containing chromium has been found to be unsanitary and unaesthetic because they pollute the soils around dumpsites. This is due to the fact that waste dump leachates are transported and distributed to the surroundings by a variety of environmental factors, such as rainfall or spread into the adjacent river system by groundwater flow or chemical reactions such as biodegradation, adsorption, hydrolysis, dissolution, dilution, partitioning and precipitation [6,7].

Edible vegetable crops grown at chromium contaminated sites take up and accumulate chromium at concentrations that are potentially toxic to human health [8]. Chromium has been reported to be the second most common heavy-metal contaminant in groundwater, soil and plants [9]. In addition, chromium(III) ions are known to be partially soluble in the soils after complexing with organic matters. At high concentrations, it creates potentially toxic environments for plant growth, causing stunted shoots, poor root development and leads to leaf chlorosis, tissue necrosis, decreased enzyme activity, membrane damage, diminished photosynthesis and changing of chloroplasts [10]. The hexavalent form of Cr is one that is biologically toxic and to date, there are no signs indicating any potential biological role it plays in plants. Its complex electronic chemistry has been a major hurdle in disentangling its toxicity mechanism in plants. Therefore, the hazardous effects of Cr are primarily dependent on metal speciation, which determines its uptake, translocation and accumulation in roots and edible portions [11]. The presence of Cr contamination in soil is toxic to edible crops as reported by Soundari and Sundaramoorthy [12]; the increasing chromium concentrations also interfered with the biochemical parameters, such as protein, sugars and amino acid contents, significantly in the plant as they decreased in all different concentrations of Cr treated soil as compared to control.

Cr affects the plants indirectly by replacing essential nutrients at cation exchange sites. The high concentration of Cr in soils causes several adverse effects on vegetables and, in the long run, affects human health. For example, Gupta et al. [13] reported that Cr contamination in marketed vegetables in Hong Kong was found at 0.56%.

This study thus aimed at evaluating the levels of Cr species that can be accumulated by *Spinacia oleracea*, *Amaranthus dubius Thell*, *Phaseolus vulgaris*, *Cicer arietinum* and *Vigna angularis* as they germinated and grew in Cr(VI) spiked soils. The study also aimed at assessing which vegetables were consistently able to germinate in the highest concentration to determine how much they bioaccumulate and translocate in different parts of their tissues (root, stem and leaf), and lastly, to estimate human health risk indices through their consumption. The increase in unplanned settlements and urban agriculture around tannery dump sites could increase the risk of cancer associated with the consumption of edible vegetables from contaminated sites. Assessing health risks could contribute to proactive mitigations.

## 2. Materials

### 2.1. Equipment

Various equipment was used for analysis. Total chromium concentration in soil and plants was determined using Agilent 700 series inductively coupled plasma optical emission spectroscopy (ICP-OES) using expert II Varian ICP expert software (Perkin Elmer, Inc., Waltham, MA, USA). The instrumental parameters used for the ICP-OES lines were power (1.5 kW), plasma flow (15 L/min^−1^), auxiliary flow (1.5 L/min^−1^), nebulizer flow (0.75 L/min^−1^), pump rate (15 rpm) and replicates 3. Cr was analysed using line (267.7Λ in nm).

Cr(VI) concentrations were analysed with a Lambda 650 Perkin-Elmer UV-visible spectrophotometer (Perkin-Elmer, Inc., Waltham, MA, USA). The spectrophotometer was first scanned for lambda mark peaks between 400 and 800 nm with blanks and samples and then calibrated with standards ranging from 0.1 µL to 4 mg/L while samples were quantified using a UV winlab-run-wavelength quant Lambda 650S-Perkin Elmer at 540 nm, with 2.00 nm slit aperture and curvete of 10,000 mm. The regression line was determined after calibration and had r^2^ = 0.999.

The soil-water suspension was homogenised by vigorous shaking using a mechanical shaker from Lab connections (St. Augustine, FL, USA). The Accsen multi-parameter probe from XS instruments (Carpi, Modena, Italy) was used for measurements of pH/EC. A model Defy DMO 350 oven from Defy Appliances (Pty) Jacobs (Durban, South Africa) was used for drying soil/plant samples. The dried plant materials were ground with a milling machine Knifetec 1095 sample mill, from Adendorff machinery mart, (Krugerdrop Johannesburg, South Africa). Total organic carbon (TOC) was measured using a spectrophotometer SPEKOL 1500 from Analytik Jena AG, (Jena, Germany). Soil hydrometer, ASTM 152H from Gilson company Inc (Lewis Center, Orange Township, OH, USA) was used for particle size analysis.

### 2.2. Soil Sampling

The soil samples were collected from the University of South Africa horticulture department, and characterised immediately after collection for the physicochemical parameters. An amount of 0.5 kg of the samples was pre-treated and pH. Electro Conductivity (EC) was analysed using a multi-parameter probe, while TOC was analysed using a spectrophotometer SPEKOL 1500. The moisture content was analysed with an oven while the soil particle texture was analysed using Boyoucos hydrometer techniques. Ch_T_ was analysed using an ICP-OES.

### 2.3. Sampling and Preparation of Seeds

The seeds of *Amarantha dubius Thell* and *Cicer arietinum* used in this study were collected randomly within Dogbone tannery dumpsite in Kenya. Seeds of *Spinacia oleracea*, *Vigna angularis* and *Phaseoulus vulgaris* were purchased from Agricol, a licenced seeds dealer in South Africa. These seeds were identified and named scientifically at herbarium section of the University of South Africa horticulture department. The seeds of *Cicer arietinum* and *Amarantha dubius Thell* from the field were packed into sterile polythene bags while *Spinacia oleracea*, *Vigna angularis* and *Phaseoulus vulgaris* seeds came with their sealed packages. The seeds of *Amarantha dubius Thell* and *Cicer arietinum* were sun-dried. Before planting, for the seeds in soil packed plastic containers in the greenhouse, the seeds’ surface were sterilised to prevent fungal and bacteria contamination by first soaking them in 30% bleach for 30 min. The seeds were then rinsed with Millique (MQ) water four times before being soaked in MQ water for 30 min. Seed imbibition (hydration) was done with MQ water overnight.

### 2.4. Preparation of Stock Solution

A 1000 mg/L stock solution of chromium was prepared using 2.9583 g of potassium dichromate in deionised water according to the procedure reported by Soundari and Sundaramoorthy [12] and Sundaramoorthy et al. [14]. The stock solution was then serially diluted to get the standard working solutions of desired chromium concentrations.

### 2.5. Experimental Design in the Greenhouse

This study was carried out in greenhouse number 6, horticulture department, College of Agriculture and Environment Sciences at the University of South Africa, Science Campus. The site is located at Latitude: S 26° 9.501, Longitude: E 27° 54.113. The study was conducted between May and July 2018, under controlled conditions.

The experiment was arranged in a complete randomised design with triplicate for each treatment. The range of Cr concentrations used in this study was chosen based on the ranges reported in the literature by Amin et al. [15] and Fernandez et al. [16]. The air-dried soil artificially spiked with different volumes of Cr(VI) solutions, i.e., 10, 50, 100 and 200 mL, respectively, along with an untreated control, were adapted. The 10 mL and 50 mL solutions were mixed with 100 mL of deionised water to get enough solution before mixing with 0.5 kg soil. Thereafter, chromium solutions were uniformly mixed with air-dried soil and kept for two weeks to stabilise as adapted from Amin et al. [15]. Three seeds of each of the edible vegetables selected for this study were sown into the spiked soil and their controls in triplicates. A total of 675 seeds were planted in the 15 containers. The planted seeds were irrigated with 80% deionised water equally three times a week. Before and at the end of the experiments, the soil and plant samples were subjected to various analyses, as detailed below.

### 2.6. Estimation/Observation of Germination and Growth Pattern

The seed germination was monitored after every 24 h until the germination percentage and growth height was constant. For the evaluation of seedling growth, all germinated seedlings of similar type were allowed to grow within concentrations of 23, 114, 228 and 456 mg/kg chromium in the soils. During their growth period, the seeds were monitored for the germination trend, growth rate/pattern, height and physiological changes, especially the stem and leaves. The plants were harvested carefully after 56 days, washed with distilled water to remove soil particles and analysed for growth attributes, such as germination percentage and growth height.

### 2.7. Germination Percentage and Growth Height

The germination percentage (%G) is the proportion, expressed as a percentage of germinated seeds to the total number of viable seeds (675) that were tested by using the formula of Bahira et al. [17], Equation (1). The plant growth height was measured periodically. At the end of the experiment, the mean height was taken for each plant under different concentration, i.e., 0, 23, 114, 228 and 456 mg/kg and the stress tolerance index for plant height (TI_PH_) was calculated using the formula of Wilkins [18], Equation (2).

(1) $$\%G=\frac{\text{Number of germinated seeds}}{\text{Total number of planted seeds}}\times 100,$$

(2) $$\left({\mathrm{TI}}_{\mathrm{PH}}\right)=\frac{\text{Height of treated plant}}{\text{Height of control plant}}\times 100.$$

### 2.8. Determination of Total Chromium in Soils and Plants

An amount of soil weighing 1 g of the homogenised sample was put into digestion crucibles, then 9 mL of HNO_3_ was added to the sample and allowed to react before adding 2 mL of hydrogen peroxide. The solution was given time to stabilise before loading onto the microwave. The vessels were heated at 180 °C for 1 h. The digested solution was filtered using Whatman filter paper (0.45 µm). Then 1 mL of the filtrate was taken and diluted to 10 mL with deionised water. This solution was analysed for total chromium (Ch_T_).

Plant samples were first cut at root–stem–leaf junctions, respectively. The fresh weight of root, stem and leaf samples were measured using an analytical balance and recorded in gram per plant. Then plant parts were dried in an oven at 60 °C for 24 h to get constant dry weight for roots, stems and leaves. Plant materials were lyophilised and homogenised by grinding in milling machine before being digested in microwave following the same procedure adopted for soil as modified from EPA method 3052 [19]. The samples were analysed using ICP-OES.

### 2.9. Determination of Cr(VI) in Soils and Plants

A total of 0.25 g of dried soil samples were put into glass beakers. To that, 25 mL of 0.1 M Na_2_CO_3_ was added, and the mixture boiled on a hot plate for 15 min. The solutions were allowed to cool and then filtered using Whatman filter paper (0.45 µm). The filtrate was topped up to 25 mL. The solution was taken and acidified with 4 mL of 0.2 M sulphuric acid to lower the pH to ~2. This was complexed with 1 mL of diphenylcarbizide and analysed for Cr(VI) at 540 nm after calibration with prepared standards. Milled plant samples were prepared and analysed for Cr(VI) using the procedure adapted from Lesniewska and Gontarska [20]. The samples were analysed using a UV visible spectrophotometer. The concentration of Cr(III) was derived from the difference between Ch_T_ and Cr(VI) concentrations. The occurrence of Ch_T_ and Cr(VI) in plant samples necessitated the quantification of their risk on human health.

The investigation of the potential risks associated with the ingestion of Cr is widely reported in the literature [20,21,22,23,24]. It can be assessed or estimated using the bioaccumulation/bioconcentration factor (BF/BCF), translocation factor (TF), daily intake of chromium (DIC), hazard quotient (HQ) and hazard index (HI) which were calculated using Equations (3)–(7).

Data were statistically analysed and validated using STATA version 14.2. The mean values of the sampled parts of edible vegetables and controls were then subjected to Tukey’s honestly significant difference (HSD) test *t* at confidence level (*p* ˂ 0.05). Multivariate statistics in terms of principal component analysis (PCA) was performed using Xlstart statistical software [25]. The PCA was used to establish major variations and relationships among the different edible parts of vegetable species and Ch_T,_ Cr(VI) and Cr(III) oxidation states. It was used to specifically identify similar active and observable variables to provide a visual summary of the results based on the dimensionality of the original data.

Bioaccumulation/Bioconcentration factor (BF) is computed according to the following Formula (3) reported by several authors [21,25,26].

(3) $$\mathrm{BF}/\mathrm{BCF}=\frac{\left[C1\right]}{\left[C2\right]},$$

where C_1_ and C_2_ are average concentrations of metal in plant and soil, respectively.

Translocation factor (TF) was calculated by the relation in Equation (4) modified from de Sousa et al. [21] and Carbonell et al. [22].

(4) $$\mathrm{TF}=\frac{\text{Cr content in the leaf}\;(\mathrm{mg}/\mathrm{kg})}{\text{Cr content in the roots}\;(\mathrm{mg}/\mathrm{kg})}.$$

Daily intake of chromium (DIC) was estimated by the modified Equation (5) of Chaturvedi et al. [23].

(5) DIC=DIV×Cr content of vegetable,

where DIV is the daily intake of vegetable (kg/day); Cr content is the Ch_T_/Cr(VI) content of vegetables (mg/kg).

Hazard quotient (HQ) an estimate of potential hazard, was determined by Equation (6) modified from Bose and Bhattacharyya [26].

(6) $$\mathrm{HQ}=\frac{\mathrm{DIV}\times \mathrm{Cmetal}}{\mathrm{RfD}\times \mathrm{BO}},$$

where DIV is the daily intake of vegetable leaves (kg/day), (C_metal_) is the concentration of Cr_T_/Cr(VI) in the vegetable (mg/kg), RfD is the oral reference dose (RfD value for Cr is 1.5 mg/kg of body weight/day) and BO is the human body weight (60 kg for adults and 25 kg for children).

Hazard index (HI) is calculated as an arithmetic sum of the hazard quotient for each pollutant, as shown in the following modified Equation (7) of Chaturvedi et al. [23].

(7) $$\mathrm{HI}=\sum_{i=0}^{n}\mathrm{HQ}.$$

## 3. Results and Discussion

### 3.1. General Properties of Soil

Various physicochemical properties of the homogenised soil used in this study were measured and are depicted in Table 1. The textural particle size of experimental soil had 25% sand, 15% silt and 60% clay suggesting the soil to be predominantly clay. Clay soils are the most balanced and support the greatest diversity of plant life. The types of plants that grow well in clay soil include grasses, bamboo, wetland and aquatic plants, vegetables and fruit trees [27]. The environmental data of the greenhouse are shown in Table 2.

### 3.2. Effect of Chromium Concentration on Seed Germination and Growth

**Figure 1:**
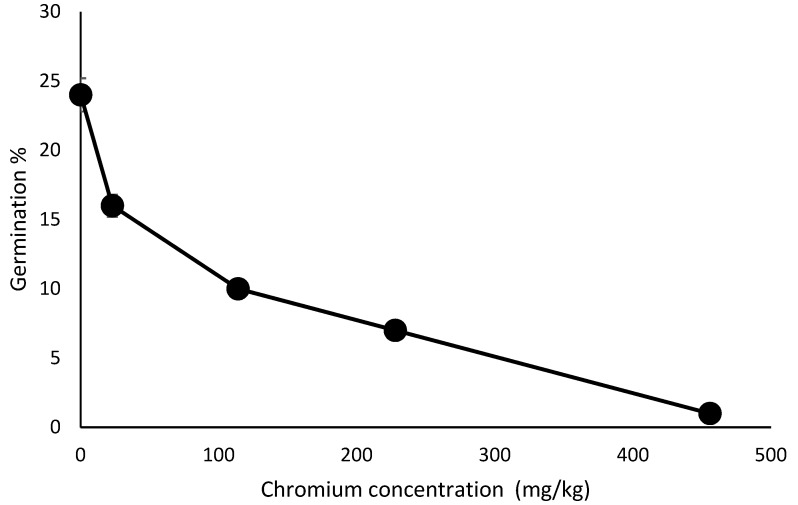
Germination percentage of plants in different concentrations of Cr(VI) along the y-axis (n = 3, SD). SD: standard deviation.

The observed results of the present study show that higher chromium concentration adversely influences the germination process of various vegetable seeds (Figure 1). Chromium treatment in soils at the level of 23 to 456 mg/kg had different effects. It was observed that 24% germination took place in the control soils but decreased to 16% in soils with concentrations of 23 mg/kg, 10% germination corresponded to soils with 114 mg/kg of chromium concentration, 7% germinated in 228 mg/kg Cr levels and 1% managed to germinate in 456 mg/kg Cr levels. Significant variations in Cr tolerance and sensitivity in terms of seed germination have been recorded in literature, according to Shahid et al. [24]. This study has established that the germination of different plant seeds is affected differently with different Cr levels (Figure 1).

The germination time in this study was prolonged to (8 ± 1 days), while the control germination was observed after (6 ± 1 days). The prolonged germination period was observed as the levels of chromium increased from 23 mg/kg to 456 mg/kg. This suggests that the seeds may have undergone secondary induced dormancy in Cr conditions before germination, and this lengthened their germination period. Eze et al. [28] stated that at a treatment level of 400 mg/kg of chromium in soil, a prolonged germination time (9 ± 1 days) was observed unlike the control (4 ± 1 days) and this implied that germination time increased with increase in chromium dosage.

**Figure 2:**
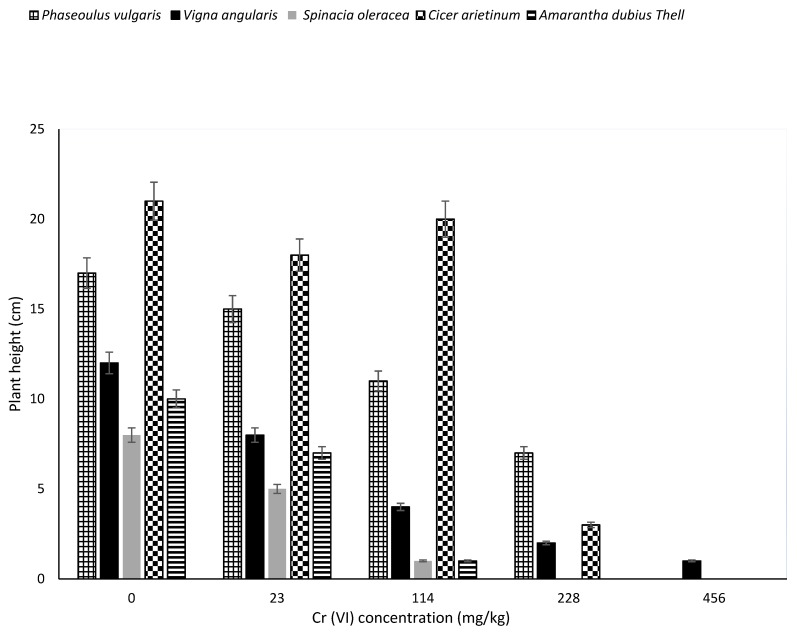
Effect of Cr(VI) concentration on different plant heights in simulated soil. (n = 3, SD). SD: standard deviation.

The Cr transported to the aerial parts or retained at the roots might have affected the physiological processes of plant growth as it contributed to their various reductions in height, as shown in Figure 2. Significant differences (*p* < 0.05) was found in plants height with increased concentration of chromium from 0 mg/kg (control) to 456 mg/kg. There was a significant difference between the height of plants in the control and those grown in soils containing varying levels of Cr (23–456 kg).

There was also a significant difference observed between Cicer arietinum and Spinacia oleracea, Phaseoulus vulgaris and Spinacia oleracea, Cicer arietinum and Amarantha dubius Thell, Phaseoulus vulgaris and Amarantha dubius Thell. The significant decrease in height could be attributed to Cr(VI) concentrations. In the 114 mg/kg Cr levels, the highest height recorded was that of Cicer arietinum, while the lowest were Spinacia oleracea and Amarantha dubius Thell. This possibly implies that Spinacia oleracea and Amarantha dubius Thell seeds growth are sensitive to a high increase in Cr(VI) concentrations at 114 mg/kg in comparison to other plants.

In 228 mg/kg levels, the maximum height recorded was that of *Phaseoulus vulgaris* and the least was *Vigna angularis*. The *Spinacia oleracea* and *Amarantha dubius Thell* seeds never germinated in 228 mg/kg simulated soil, depicting total inhibition of growth hormones. Lastly, *Vigna angularis* was the only plant that germinated and grows (up-to 1 cm) in 456 mg/kg soil. This suggest that *Vigna angularis* may be having a unique mechanism that enables it to absorb nutrients in a high chromium contaminated environment. *Vigna angularis* and other plants in this study may have used a sulphate transporter mechanism to actively transport Cr(VI) into their body cells because it is an anion which is known to be very mobile because of its negative ions, and competes with sulphate ions in the uptake due to their similarities. In addition to that, these plants may have also used the passive sorption mechanism after reducing Cr(VI) absorbed in their tissues to Cr(III). This mechanism may have involved the diffusion of Cr(III) ions across the cell wall and plasma membranes into the plant’s body for translocation to other parts. These uptake mechanisms, as suggested in this study, are also supported by literature reports [29,30,31,32].

Cr transported to the aerial parts of these plants directly impacted cellular metabolism of shoots contributing to the reduction in their height, as seen in Figure 2 of this study. This is in agreement with Sundaramoorthy and Sankarganesh [14] and Bahira et al. [17], who reported that high Cr(VI) concentrations (500 mg/kg) in soil affected shoot growth of wheat and oat. It also led to a decrease in plant height as reduced root growth was observed and, consequently, decreased nutrients and water transport to the higher parts of the plant.

**Figure 3:**
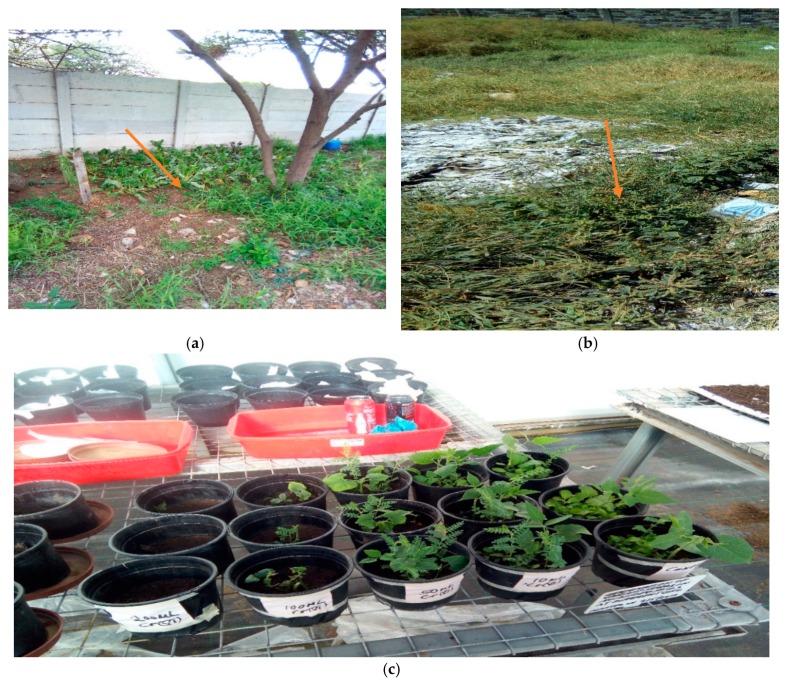
(**a**). *Spinacia oleracea* grown by tannery workers near tannery chromium wastes dumpsite in South Africa. (**b**) *Amarantha dubuis Thell* growing wildly in a tannery chromium wastes dumpsite in Kenya. (**c**) Experimental set up and germination of edible vegetables in Cr(VI) polluted soil in the University of South Africa (UNISA) greenhouse number 6.

The current study observed a significant variation in the germination and growth of edible vegetable seeds in Cr polluted soils (Figure 3c). Simulated studies using Cr(VI) as the spiking agent to investigate different crop reactions and growth have been reported by several authors [15,33,34]. Cr is considered strongly toxic because Cr(III) compounds in the soil are more or less insoluble and their ions are tightly bound to humus and clay particles while Cr(VI) is very soluble and easily passes through the plant cells into vacuoles where they combine with cations and form stable compounds which either accelerate or retard plants growth [34]. *Amarantha dubuis Thell* and *Spinacia oleracea* germinated in low concentration only while *Cicer arietinum, Phaseoulus vulgaris* and *Vigna angularis* germinated in both low and high concentration of Cr(VI). It may be possible to state here that seed coats of different plants’ impermeability and embryos’ selectivity could have affected the tolerance of chromium impacts. Those events naturally could have helped in the selection of high and low tolerant species or chromium varieties during the germination, early seedling stages and growth height in this study. These findings are in agreement with those reported by Kidd and Mart [35], who reported that higher concentrations of heavy metal significantly reduced the strength of germination as compared to the lower concentrations, which had the least harmful effect on germination. Peralta et al. [36] also found that there was no germination of spinach with applied Cr level at 320 mg/kg in the soil.

The consistent germination and significant growth of *Phaseoulus vulgaris*, *Cicer arietinum* and *Vigna angularis* in chromium contaminated soil observed in this study seem to suggest that these plants might be tolerant or have mechanisms that allow them to germinate and grow in Cr toxic environment. It could be observed that at the roots of *Vigna angularis* at the highest concentration was modified with fewer hair roots (Figure not shown) as compared to those from low concentrations and control. This could be the part of mechanism this plant applied in the roots to exclude excess Cr(VI) to its aerial parts, which made it possible for it to grow in such high concentration. Sharma et al. [37] stated that toxic properties of Cr(VI) could be reduced by oxidising agents as well as from the formation of free radicals during the reduction of Cr(VI) to Cr(III) inside the root cells of plants.

To understand practically how these plants may have applied these strategies and mechanisms to survive in Cr polluted soil, the sampled plants in this study were divided into root, stem and leaf. Then measurement involving the assessment of different levels of Cr oxidation states; Ch_T_, Cr(VI) and Cr(III) in the vegetables grown in the polluted soil was undertaken, and their occurrence is given in Table 3. Different vegetable cultivars were found to differ in their ability to take up Cr oxidation states as the occurrence varied in this study, as depicted in Table 3. This is in agreement with Fitz and Wenzel [38], who reported that different plants exhibit diverse strategies to high concentrations of Cr, such as indicators, excluders and accumulators.

The mean values of chromium oxidation states in the sampled parts of edible vegetables and their controls were then subjected to Tukey’s test at confidence level (*p* ˂ 0.05). The values were found to have statistically significant differences in their control and parts (*p* = 0.000), indicating strong significant difference between the measured variables. This could be attributed to the Cr(VI) spiked in the experimental soils which could have been accumulated by these plants differentially through their tolerance mechanisms. In the roots, *Cicer arietinum* had the highest Ch_T_ while *Spinacia oleracea* had the lowest level. *Vigna angularis* had high Cr(VI) and *Spinacia oleracea* registered the lowest. The *Cicer arietinum* had highest Cr(III) while the lowest was in *Amarantha dubuis Thell*. This depicted that *Cicer arietinum* and *Vigna angularis* tolerance mechanisms for different Cr oxidation states were superior to *Spinacia oleracea* and *Amarantha dubuis Thell* in their roots, probably from the modification of their root hairs.

The level of total Cr translocated to the stem was high in *Cicer arietinum* and *Vigna angularis* and least in *Spinacia oleracea. Vigna angularis* accumulated high Cr(VI) in the stem as *Spinacia oleracea* registered nil in the stem. *Cicer arietinum* and *Vigna angularis* had a high concentration of Cr(III) in the stem, and the lowest level was recorded in *Spinacia oleracea. Spinacia oleracea* and *Amarantha dubuis Thell* maintained their low uptake implying either efficient exclusion or poor translocation. *Vigna angularis* seems efficient in the uptake of Cr(VI), suggesting that it has a unique mechanism of transporting it in the root and stem.

The level of Ch_T_ translocated to the leaf parts was highest in *Cicer arietinum* and *Spinacia oleracea* but least in *Phaseoulus vulgaris*. *Cicer arietinum* accumulated maximum Cr(VI) in the leaf and a very low concentration was observed in *Spinacia oleracea*. However, *Spinacia oleracea* had high levels of Cr(III) in the leaf, while the least concentration was found in *Amarantha dubuis Thell*. *Cicer arietinum* and *Spinacia oleracea* transported more Cr oxidation states to the leaf, which may be due to their leaf physiology, which encouraged phytovolarisation of the Cr pollutants from the plants.

Residual chromium concentrations (Ch_T_, Cr(VI) and Cr(III)) were still detected in simulated soil at the end of the experiment, despite the effects of soil natural matrices and uptake by plants. This indicated that Cr is persistent in soil environments, and this is a source of concern. de Andrade et al. [1] stated that the risk that could be associated with Cr(VI) amendment in soils may involve the chance of Cr persistence in soil and accumulation in plants that may eventually enter into the food chain. The concentrations derived were used to assess the transfer of Cr pollutants from soil to plants’ portion using pollution indices, such as BF and TF, as monitoring tools for pollution effect on edible vegetables grown in Cr contaminated sites.

### 3.3. Bioaccumulation/Bioconcentration Factor (BF/BCF)

In this study, Cr oxidation states levels revealed significant variations in the five studied vegetables, as shown in Table 4. Significant differences (*p* < 0.05) was found in Ch_T_, Cr(VI) and Cr(III) levels between BF in the plant’s roots. Ch_T_ concentrations between *Amarantha dubuis Thell* and *Phaseoulus vulgaris*, *Spinacia oleracea* and *Phaseoulus vulgaris*, *Spinacia oleracea* and *Cicer arietinum* were significant while Cr(VI) significant levels were found between *Spinacia oleracea* and *Phaseoulus vulgaris* and *Spinacia oleracea* and *Cicer arietinum*. This suggested that these edible vegetables may have the potential to bioaccumulate and transfer chromium into their tissues. BF/BCF is an index that measures the ability of the plant to accumulate a particular metal with respect to its concentration in the soil and the root of the plant [39]. Under normal conditions, the concentration of Cr in plants is supposed to be less than 0.001 mg/kg [40]. Thus in terms of accumulation, *Cicer arietinum* could be grouped as Cr moderate accumulator plants while *Vigna angularis*, *Spinacia oleracea*, *Phaseoulus vulgaris* and *Amarantha dubuis Thell* were low Cr accumulator plants based on the Malayeri et al. [41] categorisation system. However, there were no non-accumulator and hyper-accumulator plants in this study. Instead, these plants could be grouped as phytoextractants or phytostabilisers. Since they are edible crops, this disqualifies them for phytoremediation of Cr contaminated sites.

The high values of BF/BCF confirmed that the roots are the main bio-accumulators of Cr in all its oxidation states. The highest Cr accumulation in the roots occurred due to their direct contact with Cr oxidation states in the soil. This Cr accumulation in the roots could be apportioned to that fraction of Cr ions physically adsorbed to the cell walls of the root and another fraction absorbed by the cells that were possibly immobilised in the root vacuoles. Since there was an increase in the shoot biomass, these mechanisms were suspected of having played a key role in the BF/BCF of the plants under toxic conditions in the soil. *Amaranthus* and *Spinacia* vegetables were found to have limited potential for the bioaccumulation of higher Cr concentration in their roots. That is why they were unable to germinate and grow in 228 and 456 mg/kg levels in this study. Oliveira [40] and Chandra et al. [42] explained that enhanced accumulation of chromium in the roots of *angularis* and *arietinum* species may have been due to the presence of organic acids (carboxylic acid and amino acids) in the root exudates which form complexes with chromium, thereby making them available for the uptake by roots while *Amaranthus* and *Spinacia* had low potential for accumulation of Cr. Rashed [43] reiterated that the concentration of Cr is always higher in the roots than in the shoots. These differences in Cr accumulation in different parts of the plants suggested that different cellular mechanisms of bioaccumulation of Cr took place, and this influenced Cr bioaccumulation and partitioning in these plants, thus the need to find out the quantity that was translocated from the root to the shoot.

### 3.4. Translocation Factors

**Figure 4:**
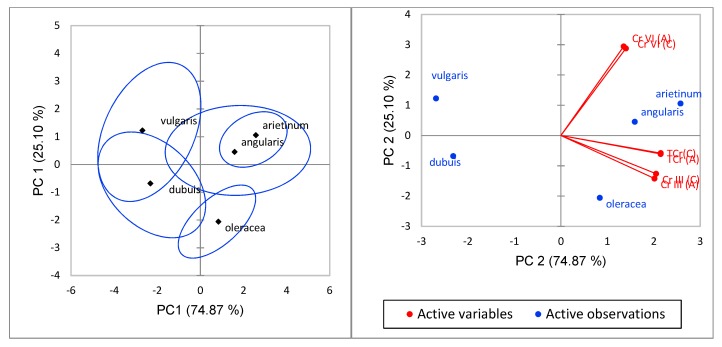
Principal component analysis (PCA) plot of the relationship between the sampled plants species (*vulgaris, dubuis, oleracea*, *angularis and arietinum*) and the relationship between concentration of Ch_T_, Cr(VI) and Cr(III) (active variables) in the stems (A) and leaves (C) of the edible vegetables (active observations) for the first two principal components obtained (PC1 and PC2).

In this study, significant differences (*p* < 0.05) were found in Ch_T_, Cr(VI) and Cr(III) levels between TF in the plants stems and leaves. The significant differences trend between Ch_T_ and Cr(VI) concentrations shown in BF were also observed in TF. To confirm the distance moved by Ch_T_, Cr(VI) and Cr(III) between these plant parts, further statistical analysis was done using principal component analysis (PCA). The relationship between the concentration of Ch_T_, Cr(VI) and Cr(III) (active variables) in the stems (A) and leaves (C) of the edible vegetables species, such as *P. vulgaris A. dubuis*, *S. oleracea*, *V. angularis* and *C. arietinum* (active observations), for the first two principal components obtained (PC1 and PC2, which account for the 74.87% and the 25.10% of the total variance, respectively) are shown in Figure 4. The active observations show that *Cicer arietinum* and *Vigna angularis* had a closer positive upper relationship than *Spinacia oleracea* that had a lower positive relationship with far distant from the two. *Amarantha dubuis Thell* and *Phaseoulus vulgaris* had a negative relationship with *Phaseoulus vulgaris* in the upper region and *Amarantha dubuis Thell* in the lower area. The active variables showed that Cr(VI) was active in the upper positive region, while Ch_T_ and Cr(III) were active in the lower positive region. This depicted Cr(VI) to be more mobile than Ch_T_ and Cr(III). *Cicer arietinum* and *Vigna angularis* were observed to be likely related in their accumulation and transfer of chromium species. Other reported studies found out that Cr is absorbed by roots from nutrient solution as Cr(III) or Cr(VI) and translocated to aerial portions and roots, but it is largely retained in the roots [44].

Given that these plants have shown bioaccumulation and translocation potential for Cr states in edible parts, they are of major health risk concern to the human population in Kenya and South Africa. Some of these plants have also been surveyed at tannery chromium dumpsites either growing wildly or grown near/at abandoned dumpsites by tannery workers in both countries, as shown in Figure 3a,b. The tendency to retain more Cr in the root > leaf > stem was seen to be common in most of the plant species, but there was also quantitative translocation among the studied plant species. This may also mean that plants with high levels of Cr in their leaves may be trying to phytovolarise the metal into the atmosphere through their leaves, as seen by yellowing from the edge towards the petal and eventually dropping off the plant after possibly translocating it and starting a new leaf growth. This is in agreement with Khan et al. [45], who reported that the maximum amount of Cr is accumulated in the roots followed by leaves and then fruits, which is close to findings in this study. According to Rashed [43] dicotyledon species, such as *Vigna angularis*, uptake and transport more Cr to shoots than monocotyledons plants, such as maize, due to differences in the rooting patterns, transpiration rates and metabolism between these two groups of plants while Cui et al. [46] oppositely found that *Amaranthus dubius* tolerated high Cr(VI) concentrations by accumulating and transferring them to aerial parts. The outcome of this study also compares to the reported findings by Oliveira et al. [47], which found that an increase in Cr concentration in the leaves may be related to higher soil Cr concentration from the spiked soil and therefore, the metal was bioaccumulated from the roots to the leaves. Lower accumulation of Cr in leaves than in roots can be related to the conservation of photosynthesis processes from toxic levels of trace elements as well, according to Zojaji et al. [48]. Thus, the levels of Cr(VI) transferred and detected in *Cicer arietinum*, *Phaseoulus vulgaris*, *Amarantha dubuis Thell* and *Vigna angularis* in the leaf portions of the plants signify high health risk to consumers.

### 3.5. Daily Intake of Chromium (Ch_T_, Cr(VI), Cr(III)) through Edible Vegetables Grown on Cr(VI) Spiked Soils

This study focused on the cultivation of vegetables on Cr(VI) simulated soils and then estimated the average daily intake of Cr by adult and child consumers of these vegetables. A significant difference (*p* < 0.05) was observed between child and adult consuming edible vegetables with Ch_T_ and Cr(III). This shows that edible vegetables in this study may have accumulated more Cr(VI) by converting it either to Ch_T_ or Cr(III) in various concentrations, but the uptake became greater than the maximum permissible limits of World Health Organisation (WHO)/Food Agriculture Organisation (FAO) [49] for plant (0.10 mg/kg) in the spiked soil. In principle, the required amount of vegetables in people’s daily diet is supposed to be between 300 and 350 g per person’s (adult) and 200–230 g per child, as suggested by guidelines of WHO [50].

**Figure 5:**
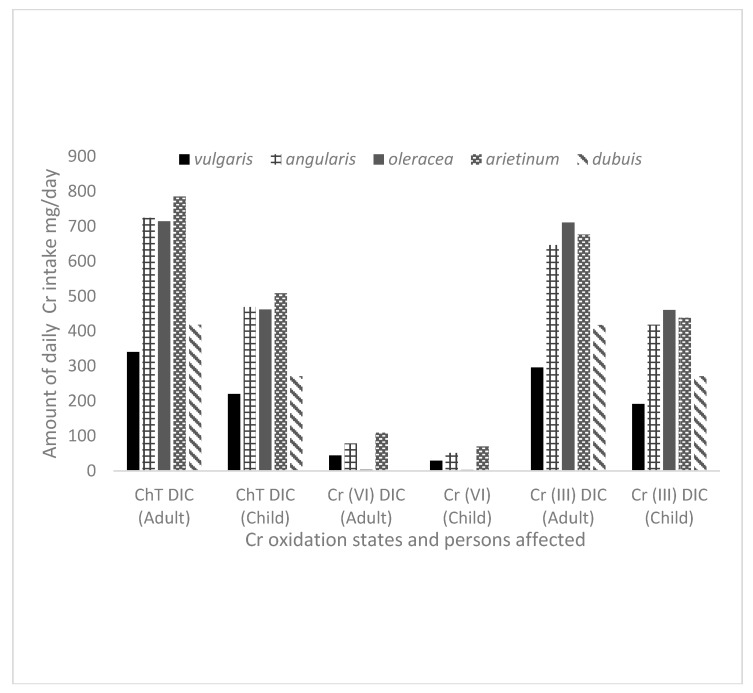
Daily intake of Chromium (DIC) total chromium (Ch_T_), Cr(VI) and Cr(III) from the leaves of different vegetables from soil spiked with Cr(VI).

Figure 5 depicts the possible daily intake of high Ch_T_, Cr(VI) and Cr(III) by humans from the edible vegetables grown in spiked soil in this study. The intake values were calculated by taking the average value of Cr oxidation states to assess the level exposure in all the five varieties of the vegetables (Table 4) and taking into account that each adult and child in Kenya and South Africa (assuming 60 and 25 kg of body weight for adult and child, respectively) consumes approximately 340 and 220 g, respectively of vegetables per day according to WHO [50]. However, the amounts in Figure 5 give a picture showing that daily intake of vegetable species was far above recommended intake levels.

In the consumption habits of local residents of Kenya and South Africa, *Spinacia oleracea*, *Cicer arietinum*, *Phaseoulus vulgaris*, *Amarantha dubuis Thell*, and *Vigna angularis* are consumed as leafy vegetables which accounts for 90% of total consumption of vegetables within the region with the remaining percentage being taken as seeds or pods [4,51]. This means that a very large population from these two countries are potentially at health risk due to exposure to these Ch_T_, Cr(VI) and Cr(III). Therefore daily intake of chromium by human consumers of these vegetables is likely to expose them to these types of clinical disorders, i.e., respiratory, carcinogenic, renal, hepatic, gastrointestinal, cardiovascular, haematological, reproductive and developmental, genotoxic and mutagenic effects [52].

### 3.6. Hazard Quotient

The results of HQ for Cr oxidation states in this study are shown in Table 5. The HQ analysis showed a significant difference (*p* < 0.05) between child and adult exposed to edible vegetables with Cr_T_ and Cr(III) from this study. For different exposure population, HQ of Ch_T_ and Cr(III) oxidation states were all above one, which suggested that the daily intake of Cr oxidation states through the consumption of *Spinacia oleracea*, *Cicer arietinum*, *Phaseoulus vulgaris*, *Amarantha dubuis Thell*, and *Vigna angularis* may likely cause adverse health effects for residents of South Africa and Kenya. Indeed, the potential non-cancer risk for *C*h_T_, Cr(VI) and Cr(III) is expressed as hazard quotient (HQ). When HQ > 1, it implies that there may be a concern for potential non-cancer effects when the chronic daily intake exceeds the threshold [39]. The levels of Cr(VI) were found to be insignificantly different, implying that consumers may be safe when they got exposed to vegetables containing it. However, according to WHO/FAO [49] guidelines for plant, the maximum permissible limits of chromium (0.10 mg/kg) were exceeded. Thus, the consumers were not safe. The safe levels were only found in *Spinacia oleracea* and *Amarantha dubuis Thell* for both adult and child. It can also be shown from Table 5 that HQ of Ch_T_, Cr(VI) and Cr(III) for children is higher than that for adults in all the plants analysed, which agrees with previous studies of Bose and Bhattacharyya [26].

### 3.7. Hazard Index

The HI method was used to assess the total of all potential health risks of Ch_T_, Cr(VI) and Cr(III) accumulation through leafy vegetable consumption for adults and children. The risk is considered unacceptable at HI > 1. The results of the five leaf vegetables were all found to be above one, which may present a risk to adults and children in terms of Ch_T_, Cr(VI) and Cr(III) exposure. HI values were observed in this decreasing order for adults and children as *Cicer arietinum > Vigna angularis > Spinacia oleracea > Amarantha dubuis Thell > Phaseoulus vulgaris*, as shown in Table 6. This suggested that the potential HI of Cr oxidation states through vegetable consumption were higher in *Cicer arietinum*, being as high as 17.2 and 27.2 for adults and children, respectively. These results implied that the potential health risk of Cr oxidation states through the consumption of leafy vegetables was higher for all vegetable types. The estimation of HI, which takes care of the chemical mixtures, is very important in assessing multiple effects of heavy metals, such as Cr. In nature, chronic low-level intake of toxic metal elements can have a negative effect on human health, and the detrimental impact only becomes known after several years of exposure [53].

The HI values of Cr oxidation states through vegetable consumption for children were higher than the values for adults in all vegetables. Therefore, Cr oxidation states are likely to contribute to the potential health risks of vegetable consumption for residents living and accessing sites contaminated with tannery Cr wastes. The HI findings in this study were lower than that of Chaturvedi et al. [23], who found HI for Cr in children’s toys at 91.9. Huang et al. [39] stated in their work that Cr speciation was of concern. This was because, according to them, health risk from Cr exposure may be overestimated if Cr(III) co-exists with Cr(VI). Cr(III) is considered essential in the metabolism of carbohydrates in animals, but high levels are equally risky under HI assessment, as seen in this study. Therefore, this study considered Cr speciation in vegetables from polluted sites and found out that their HI is a potential risk to both adults and children consuming them.

## 4. Conclusions

The present study used five species of vegetables on Cr(VI) contaminated soil in a greenhouse. *Vigna angularis* was the only vegetable that germinated at the highest Cr concentration of 456 mg/kg. *Phaseoulus vulgaris* and *Cicer arietinum* germinated at Cr concentration up to 228 mg/kg, while *Spinacia oleracea* and *Amarantha dubuis* germinated and grew at Cr concentration up to 114 mg/kg. Bioaccumulation/bioconcentrations factor of *Vigna angularis* and *Cicer arietinum* were higher than those of *Phaseoulus vulgaris*, *Spinacia oleracea* and *Amarantha dubuis*. These plants can be grouped as moderate and low accumulators but cannot be used for phytoremediation because they are edible vegetables. An adult and child who consumes these vegetables, especially *Cicer arietinum* and *Spinacia oleracea*, are likely to consume high levels of chromium above the recommended values of WHO. The HQ and HI of Ch_T_ and Cr(III) oxidation states were all above one, while that of Cr(VI) was below one. This portends carcinogenic risks to consumers, with children being more vulnerable to such risks. Therefore, this paper supports that edible vegetables cultivated in Cr contaminated soils have the potential to bioaccumulate and translocate toxic chromium compounds to edible parts, which pose a public health risk. Tannery workers usually cultivate these edibles vegetables at or near these dumpsites and consume them without adequate knowledge of the health risk associated with such food crops, thus the need for remediation of sites polluted with tannery-based Cr wastes.

## Figures and Tables

**Table 1:** Physicochemical properties of experimental soil.

Soil Properties	Average Values
pH	6.20 ± 0.05
EC (µS/cm)	42.80 ± 0.01
Total organic carbon (%)	0.98 ± 0.01
Moisture (%)	10.90 ± 0.15
Sand (%)	25
Silt (%)	15
Clay loam (%)	60
Texture Class	Clay
Ch_T_ in soil (mg/kg)	1.20 ± 0.03

**Table 2:** Environmental conditions in the greenhouse.

Temperature (°C)	Relative Humidity (g/m^3^)	Humidity (g/m^3^)	Atmospheric Humidity (g/m^3^)	Dewpoint Temperature (°C)	Radiation (J/cm^2^)
17.2 ± 4.3	74.8 ± 62.9	4.7 ± 1.5	10.6 ± 1.8	5.4 ± 3.0	35,274 ± 433

**Table 3:** The occurrence of Ch_T_, Cr(VI) and Cr(III) in root, stem and leaf of different plant species in the simulated soil. Experimental errors lower than 0.01 mg/kg have been omitted.

Name of Plant	Portion of Plant	Chromium Oxidation States (mg/kg)						P > |t| (Tukey Effect)
		Ch_T_	Control	Cr(VI)	Control	Cr(III)	Control	
*Phaseoulus vulgaris*	root	2.80 ± 0.30	0.2	0.70 ± 0.03	ND	2.10 ± 0.30	0.2	0.000
	stem	0.10 ± 0.07	0.20 ± 0.01	ND	ND	1.20 ± 0.80	0.20 ± 0.01	
	leaf	1.0	0.2	0.10 ± 0.03	ND	1.00 ± 0.03	0.2	
*Vigna angularis*	root	3.40 ± 0.60	0.2	0.90 ± 0.04	ND	2.50 ± 0.60	0.2	0.000
	stem	1.0	0.2	0.10 ± 0.02	ND	1.10 ± 0.02	0.2	
	leaf	1.80 ± 0.30	0.2	0.20 ± 0.03	ND	1.60 ± 0.30	0.2	
*Spinacia oleracea*	root	1.10 ± 0.03	0.1	0.10 ± 0.03	ND	1.00 ± 0.03	0.1	0.000
	stem	ND	ND	ND	ND	ND	ND	
	leaf	2.1	ND	ND	ND	2.1	ND	
*Cicer arietinum*	root	3.50 ± 0.50	0.30 ± 0.01	0.8	ND	2.90 ± 0.05	0.30 ± 0.01	0.000
	stem	1.0	0.20 ± 0.03	ND	ND	1.10 ± 0.01	0.20 ± 0.03	
	leaf	2.10 ± 0.20	0.30 ± 0.03	0.20 ± 0.10	ND	2.00 ± 0.01	0.30 ± 0.03	
*Amarantha dubuis Thell*	root	1.20 ± 0.01	ND	0.30 ± 0.03	ND	0.90 ± 0.03	ND	0.000
	stem	0. 10 ± 0.02	ND	ND	ND	0.1	ND	
	leaf	1.20 ± 0.03	0. 09 ± 0.06	0.20 ± 0.01	ND	1.00 ± 0.02	0.10 ± 0.07	
Chrome simulated soil	Soil	4.9	1.20 ± 0.03	1.80 ± 0.07	ND	3.10 ± 0.10	1.20 ± 0.03	0.000

ND means not detected.

**Table 4:** The Bioaccumulation Factor (BF) and Translocation Factor (TF) of total chromium (Ch_T_), Cr(VI) and Cr(III) in the different parts of vegetable plants at the harvesting stage found between Cr oxidation states and plant species (*p* ˂ 0.05).

Name of Plant	Treatment	BF	TF	
		Root	Stem	Leaf
*Phaseoulus vulgaris*	Cr_T_	0.8	0.05	0.3
	Cr(VI)	0.4	0.01	0.2
	Cr(III)	0.4	0.04	0.1
*Vigna angularis*	Cr_T_	1.0	0.30	0.7
	Cr(VI)	0.5	0.20	0.3
	Cr(III)	0.5	0.10	0.4
*Spinacia oleracea*	Cr_T_	0.3	0.02	0.2
	Cr(VI)	0.1	-	0.01
	Cr(III)	0.2	0.01	0.2
*Cicer arietinum*	Cr_T_	1.0	0.30	0.7
	Cr(VI)	0.4	0.01	0.4
	Cr(III)	0.6	0.02	0.3
*Amarantha dubuis Thell*	Cr_T_	0.3	0.04	0.4
	Cr(VI)	0.1	0.02	0.3
	Cr(III)	0.2	0.02	0.1

**Table 5:** Hazard quotients (HQ) of Ch_T_, Cr(VI) and Cr(III) to consumers of vegetables grown on soil spiked with Cr(VI).

Plant Name	Total Cr		Cr(VI)		Cr(III)	
	Adult	Child	Adult	Child	Adult	Child
*P. vulgaris*	3.8	5.8	0.5	0.8	3.3	59
*V. angularis*	8.0	12.3	0.9	1.3	7.1	11
*S. oleracea*	7.9	12.2	0.04	0.06	7.9	12.1
*C. arietinum*	8.7	13.4	1.2	1.8	7.5	12
*A. dubuis (T)*	4.6	7.1	0.007	0.01	4.6	7.1

**Table 6:** Hazard index (HI) of Cr_T_, Cr(VI) and Cr(III) in vegetables grown in soil spiked with Cr(VI) for adult and child.

HI	*Phaseoulus vulgaris*	*Vigna angularis*	*Spinacia oleracea*	*Cicer arietinum*	*Amarantha dubuis*
Adult	7.6	16	15.8	17.4	9.2
Child	11.6	24.6	24.4	27.2	14
